# Impact of the Swine flu pandemic on General Practitioner (GP) visits in Finland: sex and age differences

**DOI:** 10.1186/s12875-024-02584-1

**Published:** 2024-09-13

**Authors:** Katri Mustonen, Kaisu Pitkälä, Ossi Rahkonen, Marko Raina, Timo Kauppila

**Affiliations:** 1 Department of General Practice and Primary Health Care, University of Helsinki, Helsinki, Finland; 2 Department of Public Health, University of Helsinki, Helsinki, Finland; 3 Vantaa Health Centre, City of Vantaa, Vantaa, Finland

**Keywords:** Primary care, Swine flu, General practitioner

## Abstract

**Background:**

Swine flu might serve as a model for challenges that primary care faces during pandemics. This study examined changes in the numbers and diagnoses of general practitioner (GP) visits during and after the Swine flu pandemic in Vantaa, a Finnish city, and how GP activities recovered after the pandemic. Putative sex and age group differences were also evaluated.

**Methods:**

The study was an observational retrospective study. The monthly number of patient visits to primary care GPs by women and men in age groups 0–19, 20–64 and 65 + years was recorded before, during and two years after the Swine flu pandemic. The recorded diagnoses were also examined. The investigation period was from 2008 to 2012.

**Results:**

The numbers of monthly visits to primary care decreased from 12 324 (mean) to 10 817 in women and from 8563 to 7612 in men during the first six months of the Swine flu, returning to the original level afterwards. This decrease was thus slightly more prominent in women. However, as the size of the population increased during the follow**-**up period, the actual number of GP visits adjusted for the size of population remained at a decreased level for two years after the Swine flu. This decrease was observed especially in office-hours visits of men (from 3692 to 3260) and women (from 6301 to 5428) of 20–64 years. Swine flu did not alter the number of visits to the primary care Emergency Department. The proportion of visits with diagnostic recordings of common infectious diseases mostly decreased during the Swine flu. Only a minor impact on the distribution of recordings of chronic diagnoses was found.

**Conclusion:**

A pandemic, such as Swine flu, may decrease office-hours visits to primary care GPs. This in turn may lead to activities of primary care being adjusted downward for a long time following the pandemic. Especially the age group 20–64 years may be affected. This risk should be considered when recovery from the COVID-19 pandemic begins. Swine flu did not affect the proportion of consultations of chronic diseases, but the number of diagnoses of common infectious diseases had diminished.

## Introduction

In Finland, the health care system has encountered challenges during the COVID-19 pandemic, with about 25% of the population diagnosed with coronavirus infection [1] over a two-year period. The highest peak incidence of illness in Finland was during spring and autumn 2022 [2], but the mortality peaked again in late 2023 [3]. Because the prevalence of COVID-19 infections is currently rising again [4], it is impossible to describe how the pandemic modulates functions of post-pandemic primary health care (PHC).

To answer the question of how a pandemic may alter functions of PHC, we could study the impacts of earlier pandemics. The current pandemic is, to some extent, comparable with Swine flu, the global respiratory pandemic of 2009–2011 [5]. Both pandemics have changed either the behaviour of patients or the way that services are provided by PHC. Various aspects of the functions of primary care, such as how to monitor changes in morbidity via computerized networks [6] and provide epidemiological information on the incidence of respiratory diseases, were evaluated during the Swine flu pandemic [7]. The utilization of primary care by influenza patients has been investigated [8], as has the capability of primary care to adjust to the increased demand for consultations of respiratory disease patients [9, 10].

Alterations in primary care during the Swine flu era were thus observed. However, the recovery of use of primary care general practitioners (GPs) and diagnostics after Swine flu has not been investigated. Studies of health impacts of reductions may help health systems to reduce unnecessary care in the post-pandemic recovery [11]. Thus, experience and information regarding the recovery of the usage of services following a previous severe respiratory pandemic would be helpful in estimating future perspectives after the current COVID-19 situation, when planning the return of normal primary care functions. This study examined the changes in the numbers and diagnoses of public GP appointments during and after the Swine flu pandemic of 2008–2011 in a large Finnish city. Putative sex and age-group differences in use of primary health care office-hours services and primary health care Emergency Department (ED) were also investigated.

## Materials and methods

### Study design

This investigation is a retrospective, register-based, longitudinal follow-up study from late 2008 to early 2012. The study was performed in the public primary care system of Vantaa, the fourth largest city in Finland. Vantaa had 195 397 inhabitants at the end of 2008, and this figure rose to 203 001 inhabitants by the end of 2011. The number of women rose from 99 822 to 103 512. The corresponding increase in men was from 95 575 to 99 489. During the Swine flu the government required GPs to participate in the Swine flu vaccination programme (September 2009 to May 2010), which had an impact on the number of available office-hour GP appointments [12]. Monthly visits to GPs in primary care were studied before, during and after the Swine flu (2008–2012). Since the use of GPs by men and women may vary, the sexes were examined separately [13]. In those six months of the Swine flu when differences in use of public primary care services were observed, the office-hours visits to GPs and visits to a primary care ED doctor were separately examined in male and female patients in the age groups of less than 20 years (0–19), 20–64 years and 65 years or more (65+).

### Study measures and outcomes

The data were obtained from the Graphic Finstar patient chart system (GFS, Logica Ltd., Helsinki, Finland). The report generator of the GFS system provided monthly figures for the number of GP visits, which was the main measure analysed in the study. Visits of men and women are reported separately. The diagnoses recorded at the visit were also available. No personal data of the patients were available. Population data were provided by the statistics office of Vantaa City.

The other measure from the patient chart was the proportions of visits with various recorded ICD-10 (International Classification of Diseases 10th edition) diagnoses. The ICD-10 diagnoses were retrieved and examined at an accuracy of initial letter and first three digits. The proportions of the fifty most common diagnoses were evaluated in detail.

### Ethical considerations

The register keepers (social and health authorities of Vantaa) and the scientific ethics board of Vantaa City (TUTKE) approved the study protocol (VD/8059/13.00.00/2016). The study was implemented using the patient information system and anonymized patient data, thus without identifying the patients or physicians. All data were gathered and analysed in a manner ensuring patient and GP anonymity.

### Statistical analysis

The numbers of monthly patient visits to GPs in the same months of 2008–2009, 2009–2010, 2010–2011 and 2011–2012 were compared. The situation before the Swine flu (2008–2009) served as a control. Both absolute numbers of visits and visits/1000 inhabitants were analysed. The service providers in primary care remained the same during the follow-up, and therefore, one-way ANOVA of repeated measurements followed by t-test with Bonferroni correction was chosen as the method for statistical analysis [13]. The sex differences were compared with paired t-test. The changes in the proportions of visits with recorded diagnoses were compared against the situation before Swine flu, and Χ^2^-test was used for this analysis. SigmaPlot 10.0 statistical software (Systat Software Inc., Richmond, CA, USA) was used for the analyses.

## Results

**Fig. 1:**
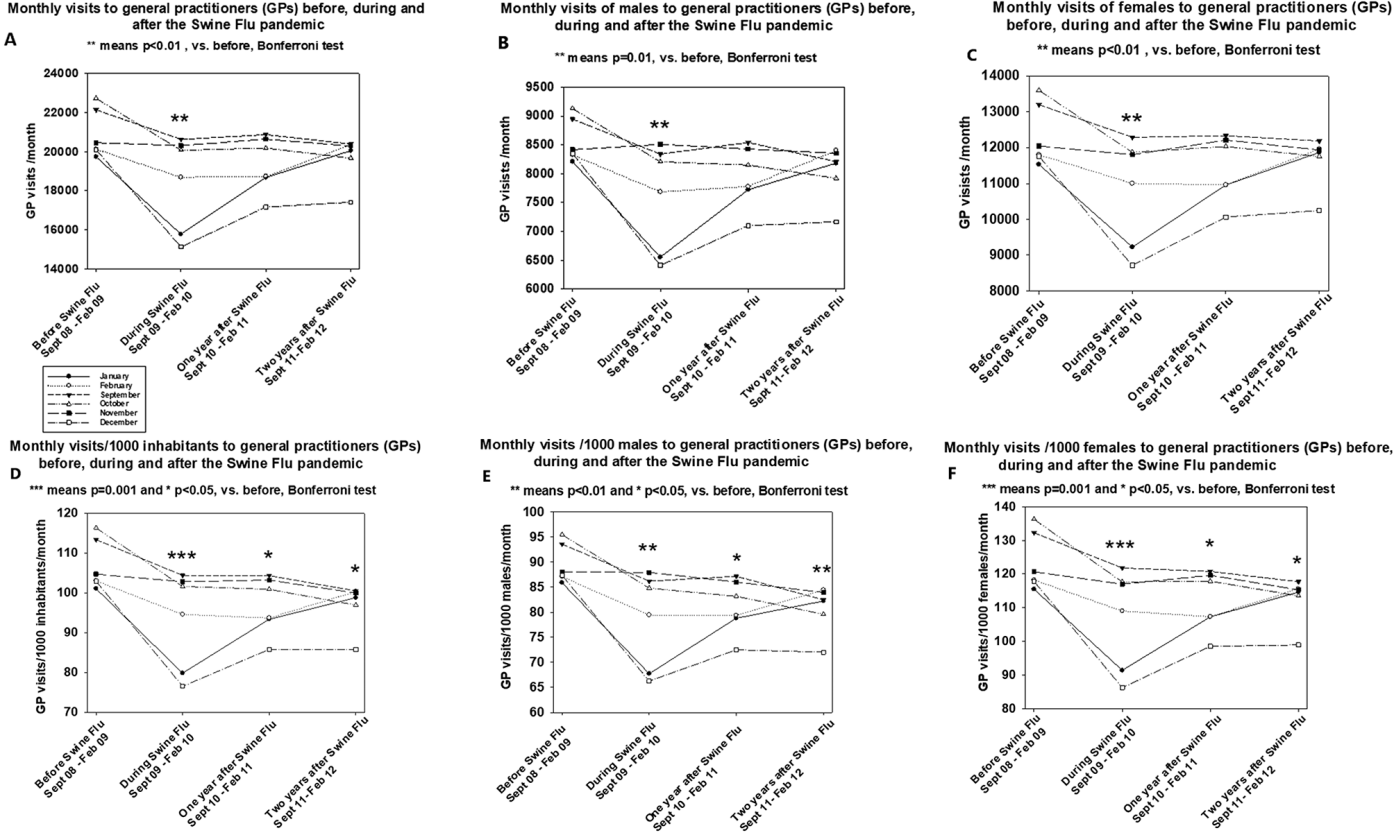
**A**-**E**. Numbers of monthly visits to GPs of Vantaa before, during and after the Swine flu pandemic. (**A**) Total visits, (**B**) visits of men and (**C**) visits of women are shown. Respective numbers of monthly visits to GPs adjusted to 1000 persons before, during and after the Swine flu. (**D**) Total visits, (**E**) visits of men and (**F**) visits of women are shown. Different symbols and lines show the development of visits to GPs in different months

The monthly number of GP visits decreased during the first six months of the Swine flu (from September 2009 to February 2010; *p* < 0.01). After the Swine flu, monthly visits to GPs returned rapidly to the original level. No reduction in monthly visits to GPs one or two years after the Swine flu was seen (Fig. 1A; Table 1).

However, adjusting for the size of the population, the reduction in monthly GP visits persisted for two years after the Swine flu pandemic (Fig. 1D; Table 1; *p* < 0.01). Reductions were seen for both men and women (Table 1, also Fig. 1B and E in men [*p* < 0.01] and Fig. 1 C and F in women [*p* < 0.01]). The reduction in monthly visits was 16.2 (SD 10.4) visits/1000 women and 10.9 (SD 7.7) visits/1000 men. This difference was statistically significant (*p* < 0.05, paired t-test). Thus, the decrease in number of visits was 5.3 monthly visits bigger (adjusted to 1000 persons) in women than the respective value in men during the Swine flu. The sex difference persisted one year after the Swine flu, as the difference between the visit rates before the Swine flu and one year after was 11.5 (SD 6.8) visits/month/1000 women and 8.4 (SD 4.6) visits/month/1000 men (*p* < 0.05). No difference was observed two years after the Swine flu: women 10.8 (SD 9.1) vs. men 8.8 (SD 6.1).

During the six most active months of the Swine flu there was no increase in absolute or relative (/1000 inhabitants) monthly primary care ED visits in any of the studied age or sex groups (Table 1). In fact, some decreases in these visits occurred in several age groups.

**Table 1:** Numbers of monthly office-hour and primary care Emergency Department (ED) visits in various age and sex groups before and one and two years after the Swine flu. Means and SD.s are shown

**Combined visits (ED + office hours)**	**Before Swine flu**	**During Swine flu**	**One year after Swine flu**	**Two years after Swine flu**
All visits, number of monthly visits	20 887 ± 1238	18 435 ± 2416*	19 381 ± 1433	19 698 ± 1154
All visits, number of monthly visits/1000 inhabitants	106.9 ± 6.3	93.3 ± 12.2**	96.9 ± 7.2*	97.0 ± 5.7*
Men’s visits, number of monthly visits	8563 ± 378	7612 ± 923*	7953 ± 533	8039 ± 461
Men’s visits, number of monthly visits /1000 men	89.6 ± 4	78.7 ± 9.5**	81.2 ± 5.4*	80.8 ± 4.6**
Women’s visits, number of monthly visits	12 324 ± 861	10 818 ± 1501*	11 428 ± 906	11 659 ± 707
Women’s visits, number of monthly visits /1000 women	123.5 ± 8.6	107.2 ± 14.9**	111.9 ± 8.9*	112.6 ± 6.8*
**Emergency Department (ED) visits**	**Before Swine flu**	**During Swine flu**	**One year after Swine flu**	**Two years after Swine flu**
Men under 20 y, number of monthly ED visits	454 ± 22	437 ± 118	337 ± 32*	358 ± 35
Men under 20 y, number of monthly ED visits /1000 persons	18.4 ± 0.9	17.6 ± 4.8	13.5 ± 1.3*	14.2 ± 1.4
Women under 19 y, number of monthly ED visits	408 ± 23	380 ± 122	318 ± 34	340 ± 43
Women under 20 y, number of monthly ED visits /1000 persons	16.9 ± 0.9	15.6 ± 5.0	13.1 ± 1.4	14 ± 1.7
Men 20–64 y, number of monthly ED visits	923 ± 56	849 ± 53	843 ± 57*	845 ± 56*
Men 20–64 y, number of monthly ED visits /1000 persons	14.9 ± 0.9	13.6 ± 0.9*	13.4 ± 0.9*	13.3 ± 0.9**
Women 20–64 y, number of monthly ED visits	1012 ± 52	997 ± 126	873 ± 57*	869 ± 81*
Women 20–64 y, number of monthly ED visits /1000 persons	16.1 ± 0.8	15.7 ± 2	13.7 ± 0.9*	13.5 ± 1.3*
Men 65 y or more, number of monthly ED visits	223 ± 35	228 ± 22	235 ± 9	263 ± 32*
Men 65 y or more, number of monthly ED visits /1000 persons	24.7 ± 3.9	23.9 ± 2.3	23.3 ± 0.9	24.2 ± 3
Women 65 y or more, number of monthly ED visits	370 ± 21	374 ± 27	376 ± 32	404 ± 52
Women 65 y or more, number of monthly ED visits /1000 persons	29.4 ± 1.6	28.2 ± 2.0	26.7 ± 2.3	26.9 ± 3.5
**Office-hour visits**	**Before Swine flu**	**During Swine flu**	**One year after Swine flu**	**Two years after Swine flu**
Men under 20 y, number of monthly office-hours visits	1807 ± 96	1510 ± 377*	1743 ± 205	1612 ± 158
Men under 20 y, number of monthly office-hours visits /1000 persons	73.2 ± 3.9	60.9 ± 15.2*	69.7 ± 8.2	63.9 ± 6.3
Women under 19 y, number of monthly office-hours visits	408 ± 23	380 ± 122	318 ± 34	340 ± 43
Women under 20 y, number of monthly office-hours visits /1000 persons	16.9 ± 0.9	15.6 ± 5.0	13.1 ± 1.4	14 ± 1.8
Men 20–64 y, number of monthly office-hours visits	3692 ± 264	3260 ± 395***	3389 ± 234**	3351 ± 229*
Men 20–64 y, number of monthly office-hours visits /1000 persons	14.9 ± 0.9	13.6 ± 0.9**	13.4 ± 0.9*	13.3 ± 0.9*
Women 20–64 y, number of monthly office-hours visits	6301 ± 614	5428 ± 843***	5810 ± 503**	5715 ± 474*
Women 20–64 y, number of monthly office-hours visits /1000 persons	100 ± 9.7	85.7 ± 13.3***	91.2 ± 7.9**	89.1 ± 7.4*
Men 65 y or more, number of monthly office-hours visits	1464 ± 138	1335 ± 191	1407 ± 145	1610 ± 126
Men 65 y or more, number of monthly office-hours visits /1000 persons	162.4 ± 15.341	140.4 ± 20.1**	139.3 ± 14.4**	148.4 ± 11.6
Women 65 y or more, number of monthly office-hours visits	2418 ± 230	2113 ± 387*	2290 ± 253	2673 ± 220
Women 65 y or more, number of monthly office-hours visits /1000 persons	191.8 ± 18.2	159.6 ± 29.2**	162.6 ± 18**	177.9 ± 14.6

*p < 0.05, ** *p* < 0.01 and *** *p* < 0.001 vs. before the Swine flu, Bonferroni-corrected t-test

Decreases in monthly number of office-hours GP visits during the first six months of the Swine flu (September-February; *p* < 0.01, RM Anova) were observed in the visits of all age groups except women less than 20 years and men 65 + years (Table 1). The decrease in this parameter persisted at one and two years after the Swine flu in the age group 20–64 years (Table 1). If the number of these visits was adjusted to number per 1000 persons, the reduction in monthly office-hour GP visits persisted till two years after the Swine flu in the same age group (20–64 years) for both sexes (Table 1; *p* < 0.01, RM Anova). There was no similar constant decrease in population-adjusted visits in the youngest (less than 20 years) and oldest (65 + years) groups of patients (Table 1).

The most prominent decreases (> 0.5%) in proportions of visits with recorded diagnoses during the Swine flu were observed in acute upper respiratory infections of multiple and unspecified sites (J06), suppurative and unspecified otitis media (H66), acute bronchitis (J20), acute sinusitis (J01), conjunctivitis (H10) and non-suppurative otitis media (H65). The proportions of these diagnoses remained at somewhat decreased levels after the pandemic (Table 2). Some respiratory symptoms, such as cough or abnormalities of breathing, remained elevated after the Swine flu. Some chronic diagnoses, such as essential hypertension, gonarthrosis, other joint disorders, not elsewhere classified and type 2 diabetes, were elevated two years after the Swine flu, but many other chronic diseases, such as depression, soft tissue diseases, back pain, other enthesopathies, atopic dermatitis, migraine, alcohol-related diseases, asthma and anxiety, were not.

**Table 2:** Proportions of visits to GPs in Vantaa with different recorded diagnoses before, during and one and two years after the Swine flu. Comparisons are made against the “before Swine flu” status

ICD-10 code	Diagnosis	% of diagnoses, before Swine flu	% of diagnoses, during Swine flu	% of diagnoses, one year after Swine flu	% of diagnoses, two years after Swine flu
J06	Acute upper respiratory infections of multiple and unspecified sites	9.6	8.6***↓	9.5*↓	8.8***↓
M54	Back pain	4.9	4.6**↓	4.6**↓	4.3***↓
H66	Suppurative and unspecified otitis media	4.4	3.1***↓	4.3	3.7***↓
J20	Acute bronchitis	4.2	3.0***↓	3.2***↓	3.2***↓
J01	Acute sinusitis	3.9	2.3***↓	3.1***↓	2.6***↓
I10	Essential (primary) hypertension	2.6	3.1***↑	2.8*↑	2.9***↑
R10	Abdominal and pelvic pain	2.5	2.7*↑	2.8***↑	3***↑
H10	Conjunctivitis	2.3	1.5***↓	2.4	1.7***↓
E11	Type 2 diabetes mellitus	2.0	**2.1**	2.0	2.3***↑
F32	Depressive episode	1.8	**1.8**	1.5***↓	1.5***↓
M79	Other soft tissue disorders, not elsewhere classified	1.5	**1.6**	1.4	1.6
M17	Gonarthrosis [arthrosis of knee]	1.2	0.9***↓	1.4***↑	1.7***↑
H65	Non-suppurative otitis media	1.2	0.6***↓	0.8***↓	0.8***↓
M75	Shoulder lesions	1.2	**1.1**	1.3*↑	1.2
J03	Acute tonsillitis	1.2	0.7***↓	0.8***↓	0.8***↓
J45	Asthma	1.2	1.4***↑	1.2	1.3
A09	Other gastroenteritis and colitis of infectious and unspecified origin	1.1	0.9***↓	0.7***↓	0.7***↓
R07	Pain in throat and chest	0.9	1.1***↑	1.1***↑	1
M53	Other dorsalgias, not elsewhere classified	0.9	0.8*↓	0.7***↓	0.7***↓
F41	Other anxiety disorders	0.9	**0.9**	0.9	0.9
S93	Dislocation, sprain and strain of joints and ligaments at ankle and foot level	0.8	0.7**↓	0.7***↓	0.6***↓
R05	Cough	0.8	1.2***↑	1***↑	1.3***↑
H60	Otitis externa	0.7	**0.7**	0.7	0.6***↓
J02	Acute pharyngitis	0.7	**0.7**	0.7	0.6
M77	Other enthesopathy	0.7	0.5***↓	0.5***↓	0.5***↓
S01	Open wound of head	0.6	**0.6**	0.6	0.7
R51	Headache	0.6	0.7**↑	0.7*↑	0.6
J04	Acute laryngitis and tracheitis	0.6	**0.7**	0.6	0.5***↓
L03	Cellulitis and acute lymphangitis	0.6	0.5*↓	0.5↓	0.5**↓
F51	Sleep disorders not due to a substance or known physiological condition	0.6	**0.5**	0.6	0.6
L20	Atopic dermatitis	0.5	**0.6**	0.5	0.5
F10	Alcohol-related disorders	0.5	**0.5**	0.5	0.6
N30	Cystitis	0.6	0.7**↑	0.6	0.7***↑
E78	Disorders of lipoprotein metabolism and other lipidaemias	0.5	**0.6**	0.5	0.4***↓
G43	Migraine	0.5	0.4**↓	0.4***↓	0.4**↓
E10	Type 1 diabetes mellitus	0.5	0.4***↓	0.4***↓	0.4***↓
R42	Dizziness and giddiness	0.5	0.6*↑	0.6**↑	0.7***↑
R50	Fever of unknown origin	0.5	**0.5**	0.4	0.4*↓
M70	Soft tissue disorders related to use, overuse and pressure	0.5	**0.6**	0.5	0.5
R06	Abnormalities of breathing	0.5	0.6***↑	0.7***↑	0.7***↑
F43	Reaction to severe stress, adjustment disorders	0.4	**0.4**	0.3***↓	0.3***↓
G44	Other headache syndromes	0.4	**0.5**	0.4	0.3***↓
I48	Atrial fibrillation and flutter	0.4	0.6***↑	0.6***↑	0.6***↑
R53	Malaise and fatigue	0.4	**0.5**	0.6***↑	0.6***↑
M25	Other joint disorders, not elsewhere classified	0.4	0.5**↑	0.5**↑	0.5*↑
S61	Open wound of wrist and hand	0.4	**0.4**	0.4	0.4
L02	Cutaneous abscess, furuncle and carbuncle	0.4	**0.4**	0.4	0.5***↑
I25	Chronic ischaemic heart disease	0.4	**0.4**	0.4	0.4
J18	Pneumonia, unspecified organism	0.4	**0.4**	0.5**↑	0.8***↑
S63	Dislocation, sprain and strain of joints and ligaments at wrist and hand level	0.4	**0.3**	0.4	0.3**↓
Z02	Encounter for administrative examination	0.4	0.7***↑	0.7***↑	0.8***↑

*p < 0.05, ** *p* < 0.01 and *** *p* < 0.001 vs. before the Swine flu. The arrows show the direction of the change

During the Swine flu no similar decreases were found in the proportions of major chronic diseases such as essential (primary) hypertension (H10), type 2 diabetes mellitus (E11), asthma (J45), atopic dermatitis (J20) or disorders of lipoprotein metabolism and other lipidaemias (E78). Analogously, the proportions of visits with mental health problem-related diagnoses, such as depressive episode (F32), other anxiety disorders (F41), sleep disorders not due to a substance or known physiological condition (F51) or reaction to severe stress and adjustment disorders (F43), were not affected (Table 2). This distribution resembled that of office-hours GP appointments (Table 3). In office hours, there was also a temporal increase in proportion of influenza diagnoses during the Swine flu, and this proportion remained somewhat elevated after the pandemic.

**Table 3:** Proportions of diagnoses in primary care office-hours practices before, during and one or two years after swine flu. Comparisons are made against the “before Swine flu” status

ICD-10 code	Diagnosis	% of diagnoses, before Swine flu	% of diagnoses, during Swine flu	% of diagnoses, one year after Swine flu	% of diagnoses, two years after Swine flu
J06	Acute upper respiratory infections of multiple and unspecified sites	10.5	9.1***↓	10.5	9.6***↓
M54	Back pain	5.0	4.7**↓	4.7**↓	4.4***↓
J20	Acute bronchitis	4.6	3.2***↓	3.4***↓	3.4***↓
J01	Acute sinusitis	4.4	3.1***↓	3.5***↓	3.0***↓
H66	Suppurative and unspecified otitis media	4.3	3.1***↓	4.3	3.7***↓
I10	Essential (primary) hypertension	3.1	3.6***↑	3.2	3.3**↑
E11	Type 2 diabetes mellitus	2.4	**2.5**	2.4	2.8***
H10	Conjunctivitis	2.3	1.6***↓	2.6**	1.8***↓
R10	Abdominal and pelvic pain	2.2	2.4*↑	2.4*↑	2.4**↑
F32	Depressive episode	2	**1.9**	1.6***↓	1.7***↓
M79	Other soft tissue disorders, not elsewhere classified	1.6	**1.6**	1.5	1.6
M17	Gonarthrosis [arthrosis of knee]	1.5	1.1***↓	1.7**↑	2***↑
M75	Shoulder lesions	1.4	**1.3**	1.5*	1.4
J45	Asthma	1.3	1.5**↑	1.4	1.4*↑
H65	Nonsuppurative otitis media	1.2	0.7***↓	0.8***↓	0.8***↓
J03	Acute tonsillitis	1.1	0.7***↓	0.8***↓	0.7***↓
M53	Other dorsalgias, not elsewhere classified	1	0.8*↓	0.7***↓	0.8***↓
A09	Other gastroenteritis and colitis of infectious and unspecified origin	0.9	0.7***↓	0.5***↓	0.5***↓
F41	Other anxiety disorders	0.9	**0.9**	0.9	0.9
R05	Cough	0.8	1.3***↑	1.1***↑	1.4***↑
M77	*Other enthesopathy*	0.8	0.6***↓	0.5***↓	0.6***↓
H60	Otitis externa	0.8	**0.8**	0.7	0.6**
J02	Acute pharyngitis	0.7	**0.7**	0.6*↓	0.6***↓
R07	Pain in throat and chest	0.7	0.9***↑	0.9***↑	0.8
S93	Dislocation, sprain and strain of joints and ligaments at ankle and foot level	0.7	0.5**↓	0.5**↓	0.5***↓
E78	Disorders of lipoprotein metabolism and other lipidaemias	0.6	**0.7**	0.5*	0.5***
L20	Atopic dermatitis	0.6	**0.7**	0.6	0.6
F51	Sleep disorders not due to a substance or known physiological condition	0.6	**0.6**	0.7	0.7
L03	Cellulitis and acute lymphangitis	0.6	**0.5**	0.6	0.5
E10	Type 1 diabetes mellitus	0.6	0.5***↓	0.4***↓	0.5***↓
M70	Soft tissue disorders related to use, overuse and pressure	0.6	**0.7**	0.6	0.6
J04	Acute laryngitis and tracheitis	0.6	**0.6**	0.6	0.5
R51	Headache	0.5	**0.6**	0.6	0.5
G43	*Migraine*	0.5	0.4**↓	0.4**↓	0.4***↓
M25	Other joint disorders, not elsewhere classified	0.5	0.6**↑	0.6*↑	0.5
F43	Reaction to severe stress and adjustment disorders	0.5	**0.4**	0.3***↓	0.3***↓
I25	Chronic ischamic heart disease	0.5	**0.5**	0.4	0.4
Z02	Encounter for administrative examination	0.5	0.8***↑	0.8***↑	0.9***↑
I48	Atrial fibrillation and flutter	0.5	0.6***↑	0.6**↑	0.6***↑
G44	Other headache syndromes	0.4	0.5*	0.4	0.4
R50	Fever of unknown origin	0.4	**0.4**	0.4	0.4
R42	Dizziness and giddiness	0.4	**0.5**	0.5	0.6***↑
M23	Internal derangement of knee	0.4	**0.4**	0.5	0.5
L02	Cutaneous abscess, furuncle and carbuncle	0.4	**0.5**	0.4	0.5
R06	Abnormalities of breathing	0.4	0.5***↑	0.6***↑	0.6***↑
I84	Haemorrhoids	0.4	**0.4**	0.4	0.4
R52	Pain, not elsewhere classified	0.3	**0.4**	0.5***↑	0.8***↑
K30	Functional dyspepsia	0.3	**0.3**	0.3	0.3
S63	Dislocation, sprain and strain of joints and ligaments at wrist and hand level	0.3	**0.3**	0.3	0.3
J11	Influenza, virus not identified	0.3	2.0***↑	0.5***↑	0.5***↑

*p < 0.05, **p < 0.01 and *** *p* < 0.001 vs. before Swine Flu, χ^2^-test. The arrows show the direction of the change

Visits due to common infectious diseases were also decreased in the ED (Table 4). Proportions of various types of otitis medias, gastroenteritis, tonsillitis and conjunctivitis were decreased also after the Swine flu. The proportion of abnormalities in breathing diagnosis remained elevated after the pandemic. The proportion of influenza diagnoses increased from 0.2 to 2.9% during the Swine flu (*p* < 0.001). This proportion had decreased to the levels (0.2%) preceding the Swine flu at one and two years after the pandemic.

**Table 4:** Proportions of diagnoses in primary care Emergency Department before, during and one or two years after swine flu. Comparisons are made against the “before Swine flu” status

ICD-10 code	Diagnosis	% of diagnoses, before Swine flu	% of diagnoses, during Swine flu	% of diagnoses, one year after Swine flu	% of diagnoses, two years after Swine flu
J06	Acute upper respiratory infections of multiple and unspecified sites	6.7	**6.2**	4.0***↓	4.3***↓
H66	Suppurative and unspecified otitis media	4.7	3.2***↓	3.9**↓	3.8***↓
R10	Abdominal and pelvic pain	4.3	**4.4**	5.3***↑	5.9***↑
M54	Back pain	4.1	**3.9**	3.9	3.7
S01	Open wound of head	2.9	**2.7**	2.9	3.4*↑
J20	Acute bronchitis	2.4	**2.1**	2.1	1.8***↓
F10	Mental and behavioural disorder due to use of alcohol	2.3	**1.9**	2.6	2.5
A09	Other gastroenteritis and colitis of infectious and unspecified origin	2.1	1.7**↓	1.6**↓	1.4***↓
H10	Conjunctivitis	2.1	1.2***↓	1.6**↓	1.2***↓
R07	Pain in throat and chest	1.9	**2.1**	2.6***↑	2.2
N30	Cystitis	1.7	**1.7**	1.9	1.7
J01	Acute sinusitis	1.6	1.3*↓	0.7***↓	0.6***↓
S93	Dislocation, sprain and strain of joints and ligaments at ankle and foot level	1.5	**1.3**	1.4	1.1***↓
S61	Open wound of wrist and hand	1.5	**1.5**	1.6	1.8
J03	Acute tonsillitis	1.5	0.8***↓	0.9***↓	1***↓
S06	Intracranial injury	1.3	0.9**↓	1.0	1.2
T74	Maltreatment syndromes	1.2	0.8***↓	1.1	0.7***↓
H65	Non-suppurative otitis media	1.1	0.6***↓	1	0.7***↓
R53	Malaise and fatigue	1.1	**1.0**	1.5***↑	2.0***↑
F32	Depressive episode	1.0	**0.9**	1.0	0.9
M79	Other soft tissue disorders, not elsewhere classified	1	1.2*↑	1.2	1.6***↑
N39	Urinary tract infection, site not specified	0.9	1.3**↑	1.2	1.2*↑
R51	Headache	0.9	1.3**↑	1.3**↑	1.1
J18	Pneumonia, organism unspecified	0.9	**0.9**	1.4***↑	2***↑
R06	Abnormalities of breathing	0.9	1.2**↑	1.2**↑	1.4***↑
R42	Dizziness and giddiness	0.8	**1.1**	1.2**	1.3***
S52	Fracture of forearm	0.8	**0.8**	0.9	1.2
J04	Acute laryngitis and tracheitis	0.8	1*↑	0.5**↓	0.5**↓
S63	Dislocation of finger	0.7	**0.6**	0.6	0.6
S62	Fracture at wrist and hand level	0.7	**0.7**	0.8	0.8
R50	Fever of other and unknown origin	0.7	**0.9**	0.8	0.7
S60	Superficial injury of wrist and hand	0.7	**0.6**	0.9	0.8
G43	Migraine	0.7	**0.6**	0.4**↓	0.5
R05	Cough	0.6	**0.6**	0.3***↓	0.5
S82	Fracture of lower leg, including ankle	0.6	**0.5**	0.6	0.8*↑
F41	Other anxiety disorders	0.6	**0.8**	0.8	0.8
A46	Erysipelas	0.6	**0.6**	0.8	0.8
L50	Urticaria	0.6	0.4*↓	0.5	0.4**↓
J45	Asthma	0.6	**0.7**	0.6	0.5
S80	Superficial injury of lower leg	0.6	**0.5**	0.6	0.5
S42	Fracture of shoulder and upper arm	0.6	**0.6**	0.5	0.6
S90	Superficial injury of ankle and foot	0.5	0.3**↓	0.4	0.4
J02	Acute pharyngitis	0.5	0.7*↑	0.8*↑	0.6
R04	Haemorrhage from respiratory passages	0.5	**0.5**	0.6	0.5
N10	Acute tubulo-interstitial nephritis	0.5	0.3**↓	0.5	0.5
R56	Convulsions, not elsewhere classified	0.5	**0.6**	0.4	0.4
H60	Otitis externa	0.5	0.3**↓	0.4	0.3*↓
R55	Syncope and collapse	0.5	**0.5**	0.6	0.6
S83	Dislocation, sprain and strain of joints and ligaments of knee	0.5	**0.4**	0.3	0.3
T78	Adverse effects, not elsewhere classified	0.5	**0.5**	0.6	0.6

*p < 0.05, **p < 0.01 and *** *p* < 0.001 vs. before Swine Flu, χ^2^-test. The arrows show the direction of the change

## Discussion

In Vantaa, the number of GP visits decreased during the years of the Swine flu pandemic (2009–2010) relative to the year before the Swine flu (2008). The decrease was more prominent in women. After the Swine flu, the number of visits to GPs recovered to the original level. However, simultaneously, the population increased, and therefore, visits per person remained at a lower level. As the GP visits decreased, the proportion of diagnosed common infectious diseases decreased compared with the previous year of the pandemic. This seemed to happen also in office-hours and ED services. The Swine flu seemed to affect most the office-hours services of the age group 20–64 years. There was no systematic increase in share of chronic diagnoses after the Swine flu.

Originally, the aim of our study was to investigate health care-seeking behaviour during the Swine flu, but we noticed that this behaviour is strongly affected by the capacity of the health care system. If GPs are not taking on their normal functions and extra effort is needed to receive an appointment due to administrative decisions of the health system, the behaviour of patients changes. This is not a new phenomenon and reducing access has been used consciously to decrease use of certain PHC services [13–15]. Nevertheless, the observed result of a decrease in the number of visits to GPs is similar to recent findings regarding the COVID-19 pandemic [16, 17]. This may be a natural consequence of reorganizing the primary care resources to tackle the exceptional circumstances of a pandemic; for example, GPs were required in preventive actions instead of working in their usual consultations [12]. The headcount of GPs increased from 94 in the year 2009 to 96 in 2010, a year after the onset of Swine flu [15]. The number of monthly visits/GP was at a level of about 170–200/month before the Swine flu, decreasing to 130–180/month during the pandemic and later returning to pre-pandemic levels [15]. No clear rebound was observed in these visits after Swine flu ended.

In previous studies, the decrease in GP services began before outbreak of the Swine flu [13, 15]. The Swine flu may have changed the functions in the primary care system, resulting in permanently decreased visits to GPs. Most likely this prolonged decrease was not planned, but the possibility of intentional cost-savings cannot be ruled out. According to earlier studies, the observed decrease persisted at least until 2014 [13, 15]. Nevertheless, it is important to understand that the stress caused by a pandemic to primary health care may have long-lasting repercussions for the functioning of the system.

The larger decrease in the number of visits by women than by men is consistent with former publications. The direct restrictions induced by Swine flu mostly affected office-hour GP activities [12]. An earlier study from Vantaa suggested that office-hour GP visits of women are more sensitive to primary health care system changes than those of men [13]. There are several former studies suggesting that primary care GP services are more often used by women than by men for various reasons. Women tend to visit primary health care more often than men when they have health issues [18–21], and they have a significantly higher mean number of visits to primary care and diagnostic clinics than men [22]. In Vantaa, this increased use of PHC by women was most prominent in the age group 18–64 years [23]. Women are also more often active in attending various health check-ups than men [24]. Thus, as women use primary care services more frequently than men, they might also be more affected by the restrictions caused by a pandemic.

It remains to be determined why management of the age group 20–64 years in office-hours primary health care remained altered after Swine flu. The number of visits showed a prolonged decrease in this age group alone. Presumably, this age group became accustomed to searching for primary care services elsewhere, e.g. the private sector, when public health care was restricted. In ED, there was no such decrease in any of the studied age groups. This is logical as emergency situations do not vanish during pandemics and must be tackled as they appear.

The impact of the Swine flu on the distribution of recorded diagnoses in visits to GPs has not previously been investigated. There seemed to be some kind of change in management of common infectious diseases, as proportions of these diagnoses decreased permanently in all parts of primary health care. Unlike the Swine flu, the COVID-19 pandemic has been reported to decrease recorded diagnoses of chronic diseases in primary health care [25, 26], and it has been suggested to lead to increased diagnostic demand of these chronic diseases in the future [26–29]. Putatively, late increases in proportions of gonarthrosis and type 2 diabetes mellitus diagnoses reflect such delays in diagnostic activities during a pandemic. It is possible that increases in the proportion of pneumonia diagnoses after Swine flu may reflect increases in latent lung diseases induced by Swine flu. Nevertheless, these changes were small and there seems to be a clear difference between COVID-19 and Swine flu in this respect. The most evident explanation for this difference lies in the differing duration and severity of these two pandemics; COVID-19 is far more severe and long-lasting than Swine flu [1–3, 7, 30]. Furthermore, the actions that PHC administrators took during Swine flu, e.g. sending some physicians to supervise vaccinations [12], were not as drastic as those taking place during peaks of COVID-19 (closing practices and restricting PHC activities due to fear of spreading the infection). Also in Finland, the measures taken during the Swine flu to limit contact and protect against virus infection were not analogous to the measures taken during the COVID-19 pandemic.

The proportion of recorded diagnoses of acute common infections decreased. This has been also observed during COVID-19 in the Netherlands [31]. It remains to be studied whether there really were fewer acute infections in the population during pandemics. Evidently, people may have decided to cope with common infectious diseases using home remedies, in an atmosphere of what they viewed as a major societal infection. Alternatively, they may have been afraid of becoming infected through a visit to a GP’s office or they may have refrained from all contact to avoid infections. Qualitative research should also be performed on this subject.

This was a retrospective study concerning PHC. As this study was purely register-based, the subjects were not aware of their participation in the study. The results reflect real clinical activity in this respect. The data are complete, but unfortunately the number of parameters available was limited. Although all public PHC visits to health centres and primary care ED were noted, these visits had no recorded diagnoses. In the present study, 80–90% of the visits had a recorded diagnosis [32]. Furthermore, there has also been a long-lasting unexplained decreased trend in the numbers of visits to GPs in Finnish PHC [33], and Vantaa is not an exception to this phenomenon in primary care office-hours [13] or in ED visits [34]. While health policies and the health system in Vantaa were not otherwise changed during the follow-up, there may have been unknown secular trends affecting the results.

As a limitation, data about possible changes in other patient characteristics or changes in ways of managing practices and diseases were not available. These factors have a considerable effect on changes in the number of visits to GPs. Data concerning these putative changes could have been obtained had there been access to individual patients’ information. This would have also allowed the following of individual patients and the expression of data per patient-years instead of using the less accurate parameter per 1000 inhabitants. We do not have information on the use of complementary private PHC, and therefore, we are unable to determine whether there was any considerable shift from the public to the private sector during and after the Swine flu pandemic. Furthermore, the official municipal description of the actions taken against Swine flu was vague [12]. We know that preparations for the pandemic started already in the latter part of September, as vaccinations began and GPs were moved from their offices to supervise the vaccinations [12]. Vaccinations were continued during the first months of the following year [12]. Thus, administrative actions taken to curb the pandemic caused a decrease in visits, and this decrease may have been even more marked than the decrease caused by the disease itself. Thus, it is truly difficult to differentiate the effect of the pandemic from the effects of administrative actions triggered by the pandemic in society. Furthermore, the public health decisions were not transparent, and the reasons behind the decisions were not disclosed. The explanations for decreased appointments to GPs may vary, but after experiencing yet another pandemic, it is crucial to note the changes that took place in the previous pandemic to ensure that the same patterns are not repeated.

ED physician services were less affected than office-hour physician services by Swine flu. This was not unexpected since staff for preventive actions were recruited from physicians working in office-hour primary care services. Furthermore, activities and demands of office-hour and primary care ED services are known to differ [13, 15, 34]. It remains to be studied why just the services of the age group 20–64 years were decreased. In principle, the Swine flu-induced decrease in GP visits should have been observed across all of the age groups simultaneously.

The COVID-19 pandemic impacted primary care in various ways [11, 35], e.g. by diminishing the diagnoses of chronic diseases [25, 26], which leads to either increased diagnostic demand of these chronic diseases in the future [26–29] or undiagnosed chronic conditions with severe outcomes to the population. Furthermore, COVID-19 decreased physical visits to PHC [16, 17], as did Swine flu. COVID-19 killed more people than seasonal influenza during winter 2023–2024 in the USA [36], while COVID-19 mortality was at its peak in Finland in fall and winter 2023 [37]. The prevalence of COVID-19 seems to be on the rise again in Finland [4] and thus, we are not yet in a post-pandemic era. Experiences from a former pandemic may therefore be of use when adapting PHC to the period following COVID-19. Yet, various questions remain to be studied and COVID-19 may provide a good opportunity to learn more about actions of PHC. Are all patient groups equally affected by a pandemic? How long can a pandemic cause upheaval to the normal functions of PHC? Does a pandemic modify the functions and services of primary care permanently and if so, how? It is also worth examining how patients alter their behaviour during a pandemic. To avoid unnecessary shortages in the resources of PHC, the lessons from the Swine flu should be considered when faced with the post-COVID era.

## Conclusion

The Swine flu pandemic decreased the number of GP visits overall, but the decrease was more prominent in women than in men. The age group 20–64 years was most affected. Although the crude number of visits to GPs recovered soon after the pandemic, there seemed to be a more long-lasting decrease in relative supply of GP work, when growth of the population was considered. Management of common infectious diseases was strongly affected, and their proportions in diagnoses were markedly reduced. Swine flu had less long-lasting systematic effects on management of chronic diseases. This finding should be considered as primary care prepares to recover from the COVID-19 pandemic.

## Data Availability

The data for the study is obtained from electronic patient chart system of Vantaa (Graphic Finstar); the authors do not have permission to share the original data with personification, but anonymized raw data is available from Katri Mustonen by request.
